# The emerging role of the exosomal proteins in neuroblastoma

**DOI:** 10.3389/fonc.2024.1414063

**Published:** 2024-06-19

**Authors:** Swapnil Parashram Bhavsar, Martina Morini

**Affiliations:** ^1^ Pediatric Research Group, Department of Clinical Medicine, Faculty of Health Sciences, UiT - The Arctic University of Norway, Tromsø, Norway; ^2^ Laboratory of Experimental Therapies in Oncology, IRCCS Istituto Giannina Gaslini, Genova, Italy

**Keywords:** exosomes, metastatic progression, proteomic analysis, neuroblastoma-derived exosomes, tumor microenvironment, exosomal proteins, biomarkers

## Abstract

Exosomes are a subclass of extracellular vesicles shown to promote the cancer growth and support metastatic progression. The proteomic analysis of neuroblastoma-derived exosomes has revealed proteins involved in cell migration, proliferation, metastasis, and in the modulation of tumor microenvironment - thus contributing to the tumor development and an aggressive metastatic phenotype. This review gives an overview of the current understanding of the exosomal proteins in neuroblastoma and of their potential as diagnostic/prognostic biomarker of disease and therapeutics.

## Introduction

### Neuroblastoma

Neuroblastoma (NB) represents the most common extracranial solid tumor in children (1). It shows a clinically heterogenous behavior, ranging from a highly metastatic disease with poor prognosis to spontaneous regression or differentiation into benign ganglioneuroma (2). The *MYCN* oncogene is most frequently amplified in NB and its overexpression is associated with poor prognosis, advanced disease, and metastasis (3, 4). About 70% of the patients diagnosed with NB exhibit metastatic disease (5) and around 15% of all pediatric cancer-related deaths are due to NB (6). Studies have shown that distant metastases remain the leading cause of NB mortality in children (7), however the molecular mechanisms underlying tumor metastasis is not fully understood. In recent years, research into exosomes have provided important insights into the fine regulation of metastasis in multiple cancers (8–10).

### Exosomes and their cargo

Exosomes, originally described around 30 years ago (11, 12), are a subclass of the extracellular vesicles (EVs), formed by endosomal pathway and secreted by a variety of cell types. These are small, membrane-microvesicles (30 – 150 nm) which contain functional biomolecules like DNA, RNA, proteins, and lipids. Their primary function is to mediate intercellular communication (13, 14). The biogenesis of exosomes is a complex process involving multiple proteins and metabolites, reviewed in detail elsewhere (15, 16).

There are four very important features of exosomes that have created an explosion of interest in the scientific community (17). First, exosomes are important means of cell-to-cell communication (18, 19). Second, they can horizontally transfer their cargo, containing important functional biomolecules like proteins and nucleic acids, to the recipient cells (20–22). Third, they are a contributing factor in the development of several diseases (23). Finally, they could act as vectors for drug delivery (24). All these hallmarks of exosomes could be exploited for their development as tools of disease diagnosis, prognosis, and therapeutics.

Studies aimed at identifying and investigating tumor-derived exosomes in modulating cancer cell invasion have contributed to provide important insights into the metastasis process and possibility of therapeutic intervention. A seminal study by Hoshino and colleagues has suggested that tumor-derived exosomes could prepare pre-metastatic niche (PMN) - a ground well prepared for cancer cells to metastasize, to facilitate organ-specific metastasis. They demonstrated that exosomes from the mouse and human tumor cells fuse with the favored resident cells at their predicted destination. For example, human lung-tropic tumor cells fuse specifically with lung fibroblasts and epithelial cells. They thus show that tumor-derived exosomes taken up by the organ-specific cells prepare the PMN. Further proteomic analysis of exosomes revealed differential expression of integrin proteins associated with either lung- or liver-metastasis. Finally, they propose that exosomal protein signature could identify cancer patients at risk for metastasis (25). The pro-metastatic role of exosomes has also been demonstrated for an aggressive brain tumor as glioblastoma (GBM) (26). Thus, GBM-derived exosomes can induce the malignant transformation of stromal cells, promoting tumor progression (27). Moreover, it has been reported that exosomes mediate the intercellular trafficking of PTEN, whose absence in the nucleus has been associated with tumor aggressiveness (28). A key factor facilitating the internalization of PTEN-containing exosomes is Ndfip1, which is repressed in GBM, preventing the accumulation of PTEN in the nucleus and thus, leading to tumor proliferation (29). Besides favoring tumor progression, GBM-derived exosomes are directly involved in chemoresistance acquisition. Specifically, glioma stem cells (GSCs), which represent a small proportion of GBM tumors, express an adenosine nucleotide that confers resistance toward pharmacological treatment. Exosome-mediated transfer of adenosine-producing enzymes can induce chemoresistance in recipient cells (29). GBM also takes advantage of exosomes as a therapeutic escape mechanism: drug internalization within exosomes can hinder their efficacy against the tumor (30).

Studies in different cancers have shown that exosomal proteins play a significant role in inducing angiogenesis and vascular permeability (31, 32), remodeling of extracellular matrix (ECM) and epithelial to mesenchymal transition (EMT) regulation (33), facilitating the formation of the PMN (34, 35), and mediating drug resistance (36). For example, Maji et al., studied the proteomics data from the ExoCarta database. They found a very higher expression of Annexin II (Anx II) in the exosomes and its positive correlation with breast cancer cell invasiveness which prompted them to investigate the biological role of exosomal Annexin II (exo-Anx II) in cancer. Significantly higher expression of Anx II was observed on malignant breast cancer cells-derived exosomes compared to the vesicles derived from normal cells, confirming that Exo-Anx II correlates positively with the aggressiveness of breast cancer cells. In addition, authors showed the role of Exo-Anx II in promoting tPA-dependent angiogenesis. Moreover, using the exosomes derived from the organ-specific metastatic variant cell lines, MDA-MB-831 (brain metastatic) and MDA-MB-4175 (lung metastatic) they showed that intravenous injection of exosomes in mice created a favorable microenvironment for metastasis and led to organotropism of the breast cancer exosomes. Thus, they propose that, given the role of Exo-Anx II in angiogenesis and metastasis, it could function as potential biomarker or therapeutic target for the diagnosis and treatment of breast cancer metastasis (37).

In another study, to understand the molecular mechanisms of colorectal cancer (CRC) metastasis, Ji and colleagues, compared the proteomic profiles of exosomes derived from human SW480 (primary) and SW620 (lymph node metastatic variant of SW480) isogenic CRC cell lines They identified 941 proteins in SW480- and 796 proteins in SW620-derived purified exosomes. Critical analysis of these differentially expressed exosomal proteins revealed selective enrichment of metastatic regulators (S100A8, S100A9, TNC and MET) and signal transduction molecules (JAG1, SRC, TNIK, EFNB2) in metastatic SW620 cells relative to primary SW480 cells. Moreover, signal transduction components (Met, Src, GRB2), lipid raft-associated components (FLOT1, FLOT2, CAV1, PROM1) and a key regulator of cytoskeletal rearrangement and cell spreading (TNIK-RAP2A complex) was uniquely expressed in SW620-derived exosomes. Their findings thus propose the unique role of exosomes in a crosstalk between tumor and stromal cells in the TMN for the initiation and progression of cancer (38).

In a very interesting study, Melo and colleagues found that pancreatic cancer cell-derived exosomes were enriched with glypican-1 (GPC1) protein and could act as a potential diagnostic and screening tool to detect early stages of pancreas cancer. Exosome protein profiling revealed 48 unique proteins in pancreatic cancer cells (MDA-MB-231)-derived exosomes. GPC1 was specifically detected only on cancer exosomes. Further experiments on the nude mice with orthotopic MDA-MB-231 tumors revealed that GPC1^+^ circulating exosomes (crExos) are derived from cancer cells in tumor-bearing mice. Next, to prove GPC1^+^ crExos as a biomarker, they isolated crExos from patients with breast cancer, pancreatic ductal adenocarcinomas (PDAC) and healthy donors. Analyses of the crExos revealed that breast and PDAC patients had higher levels of GPC1^+^ levels than healthy individuals. More importantly, the levels of GPC1^+^ crExos correlated with the tumor burden and survival in mice and patients with pancreas cancer (39).

In yet another interesting study, Costa-Silva and colleagues demonstrated that PDAC-derived exosomal migration inhibitory factor (MIF) primes the liver for metastasis and could be an important prognostic biomarker for the development of PDAC liver metastasis. MIF is highly expressed in PDAC-derived exosomes and that successful blocking of MIF prevents liver PMN formation and exosome-induced PDAC metastasis. In this study, they delineate the sequential steps in the formation of liver PMN induced by PDAC-derived exosomes. The PDAC-exosomes (with high levels of MIF) selectively target and activate Kupffer cells in the liver leading to induction of TGFβ secretion, which in turn activates hepatic stellate cells leading to formation and upregulation of fibronectin. Subsequently, bone marrow-derived macrophages and neutrophils bind to fibronectin enriched hepatic sites which finally leads to PMN formation (35).

Exosomal proteins, due to their high stability, long half-life, and direct action on their target - without the need of getting transcribed or translated, are of significant interest. Characterization of exosomal proteins could provide valuable information on exosomal origin, targeting, and their cellular effects. In addition, qualitative, and quantitative data of these proteins generated by means of advanced mass spectrometry techniques with improved data collection or analysis methods could provide accurate information to serve them as biomarkers for disease diagnosis, prognosis, and therapeutics (40, 41). Thus, comprehensive understanding of the effects of exosomal proteins on cancer biology could provide important insights into their role and function. Given the very few reports in the context of NB, it is essential to explore the potential of exosomal proteins as a biomarker and driver of tumor metastasis.

### Exosomal proteins in neuroblastoma

Using the most relevant scientific publications available on the PubMed website, here, we discuss the role of exosomal proteins in NB. We searched the keywords, “exosomes proteins neuroblastoma” in the PubMed search option (https://pubmed.ncbi.nlm.nih.gov/) in February 2024, which produced 84 results. Although 84 results were displayed, 81 entries were specific studies on either proteins or exosomes or NB. There were only three studies which had investigated the actual role of the exosomal proteins in NB. Since the focus of the review was ‘exosomal proteins in neuroblastoma’, accordingly, we selected three very relevant scientific articles (**Table 1**) for further analyses and discussion.

**Table 1:** The articles investigating the role of the exosomal proteins in neuroblastoma.

Model system	Protein profiling	Functional effect of exosomal proteins in neuroblastoma	References
Cell lines	Exosomes	Extracellular matrix assembly and adhesion, neuronal development, amoeboidal cell migration and mitochondrial activity	(42)
Cell lines	Exosomes	defense response, cell differentiation, cell proliferation and modulation of tumor microenvironment	(43)
Plasma	Exosomes	Tumor development and aggressive metastatic phenotype	(44)

**Figure 1:**
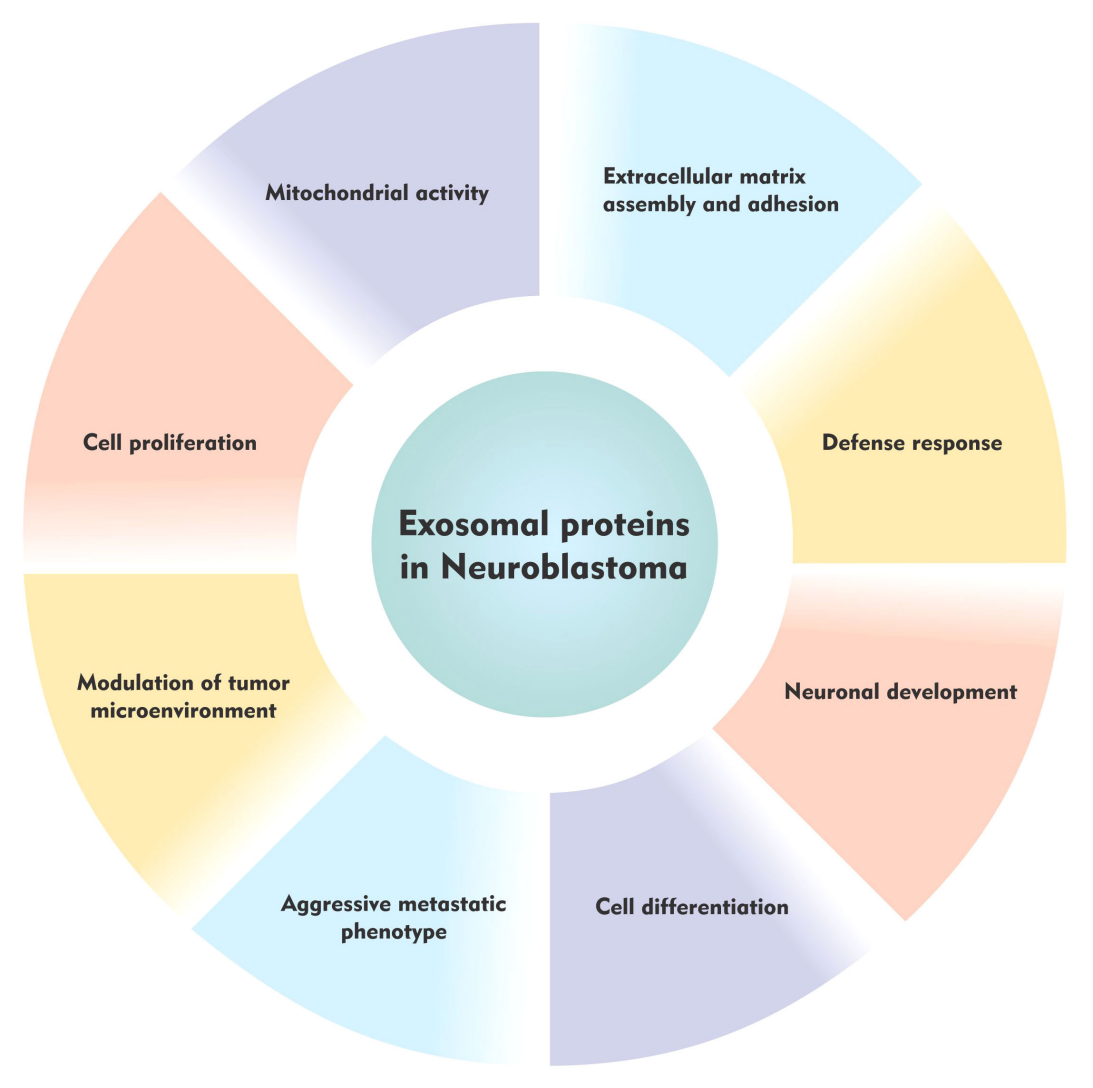
The role of the exosomal proteins in neuroblastoma.

In 2013, Marimpietri and colleagues, were able to successfully characterize for the first time the protein content of exosomes isolated from human NB cell lines: HTLA230, IMR32, SH-SY5Y, and GI-LI-N. Dynamic light scattering analysis (DLS) and transmission electron microscopy (TEM) determined the size and the exosome like cup-shaped morphology of the isolated vesicles. Zeta-potential of the exosomes suggested good vesicle stability. Furthermore, proteomic analysis of NB-derived exosomes was done by the two-dimensional liquid chromatography separation and mass spectroscopy (LC-MS/MS) analyses. Next, validation of the identified proteins was done by flow cytometry. Out of the 390 proteins identified using multidimensional protein identification strategy, almost 80% were exosome-associated and listed in ExoCarta, which is a manually curated database of proteins, lipids and RNA molecules identified in exosomes and freely available online for exosome analyses (45–47). The remaining 20% of unique NB-derived exosomal proteins likely represents a ‘signature’ of cells of neuroblastic origin. Authors concluded that NB cell-derived exosomes express a discrete set of proteins which play an important role in the modulation of tumor microenvironment (TMN) (**Figure 1**) and may represent potential tumor biomarkers (42).

Similarly, Colletti et al., in 2017 performed another study to identify an exosomal signature particularly associated with NB metastatic bone marrow dissemination. Exosomes were isolated from two NB cell lines (IGR-NB8 and IMR-32) derived from the primary tumor sites, five cell lines (LAN-1, IGR-N91, SH-SY5Y, SK-N-SH, and SKNBe2c) derived from the bone marrow metastatic site and one cell line (CHLA-255) derived from the brain-metastatic site. Scanning electron microscopy (SEM), and NanoSight particle-tracking analysis (NTA) were used to determine the size and the cup-shaped morphology of the NB cell line-derived vesicles. In addition, western blot analysis was employed to confirm the enrichment of exosome markers (TSG101 and CD63) in the extracellular vesicle samples. Subsequently, proteomic analysis of exosomes derived from the primary and metastatic cell lines performed by the very sensitive Liquid Chromatography Mass Spectrometry (LC-MS/MS) technique revealed a total of 5086 proteins. Interestingly, the comparison among the exosomal proteins from the different cell lines revealed proteins exclusively contained in exosomes from the primary or bone marrow metastasis exosomes. Proteins found in the primary tumor were associated with neuronal development, extracellular matrix assembly and adhesion, whereas those found in the bone marrow metastasis exosomes were upregulated and involved in cell motility and metabolism (**Figure 1**). This study proposed that exosomal proteins derived from NB exosomes could serve as potential tumor biomarkers (43).

Until now, proteomics studies on NB-derived exosomes were done *in vitro* using cell lines (42, 43) but there was no study carried out on patient-derived samples - thus limiting the translational applicability of exosomal cargo to clinical settings. Taking this into consideration, very recently, a study by Morini and colleagues has reported for the first time, that the exosomal proteins identified in the plasma of NB patients are associated with tumor phenotype and disease stage. The main objective of the study was to identify novel non-invasive diagnostic or prognostic biomarkers in NB. Therefore, exosomes were isolated from the plasma of low-risk and high-risk NB tumors and age-matched control subjects using the exoRNeasy Serum/Plasma midi kit. Protein cargo of the exosomes was then profiled by high-resolution mass spectrometry coupled with liquid chromatography. This analysis led to the identification of 458 exosomal proteins. They compared the exosomal protein expression profiles between High/Low risk NB patients and age-matched healthy controls. Results showed that exosomal protein expression profiles were different in NB patients vs control subjects and in high- vs. low-risk cases. In the former comparison, modulated exosomal proteins were mainly involved in cell proliferation, ECM interaction, inflammation, and immune response. In the latter comparison, pathway analysis of differentially expressed exosomal proteins showed an enrichment of cell migration, angiogenesis and EMT process, thus suggesting that exosomes of NB patients can contribute to NB tumor development and to the acquisition of the aggressive metastatic phenotype (**Figure 1**). Specifically, a high expression of Neural cell adhesion molecule (NCAM) and Nucleolin (NCL) and a low expression of Lumican (LUM) and Vasodilator-stimulated phosphoprotein (VASP) was reported in NB patient’s vs controls, showing a strong diagnostic power. Moreover, a high expression of Myosin heavy chain 9 (MYH9), Fibronectin-1 (FN1) and Latent-transforming growth factor beta-binding protein 1 (LTBP1), and a low expression of Calreticulin (CALR) and A-kinase anchor protein 12 (AKAP12) was observed in high-risk patients derived exosomes compared to low-risk cases, highlighting their prognostic power. Importantly, the diagnostic and prognostic value of the identified modulated exosomal markers improved when considering the combination of the proteins (44).

### Clinical applications of exosomes

Circulating exosomes could carry biological cargo representing the current physiological conditions (48). Given the significant role of the exosomes in normal and pathophysiological conditions, they could serve as important biomarkers of disease detection and monitoring. Major applications of exosomes include them as biomarkers (49), drug delivery vectors (50), for exosome therapy (51) and as cancer vaccines (49, 52). Multiple studies have reported the diagnostic application of exosomes in various disease conditions, including and not limited to cancer (53–57). The role of exosomes as a source of cancer biomarkers has been deeply explored because the early detection of tumor is essential to obtain the highest therapeutic efficacy (58). Expression levels of the exosomal miRNAs and exosomal proteins have been associated with multiple cancer types, thus providing a diagnostic or prognostic potential for cancer detection and management (59–62). Moreover, owing to their efficiency in delivering functional cargo to the recipient cell with minimal immune response, exosomes are actively explored as therapeutic agents (63). In this regard, several companies have now demonstrated the therapeutic potential of exosomes in pre-clinical and clinical studies, as reviewed in detail elsewhere (48, 49). Currently, liposomes are the most common nano-carriers employed for drug delivery. Exosomes could provide advantages over liposomes, because they are endogenous vesicles and their surface proteins and receptors may facilitate tissue and cell-specific targeting (64). Specifically, exosomes derived from mesenchymal stem cells (MSCs) have been investigated for cancer treatment, as they can be engineered to carry molecules that increase sensitivity to chemotherapy and may be useful for stimulating damage repair upon radiotherapy (65). Despite several companies have now demonstrated the therapeutic potential of exosomes in pre-clinical and clinical studies (48, 49), further investigations are still needed to optimize exosomes loading with therapeutic agents and to identify a suitable method for exosomes large-scale production (49). Finally, exosomes gained attention because of their ability to facilitate antigen presentation and, thus, induce an immune response. Indeed, dendritic cells-derived exosomes of melanoma patients were loaded with tumor-specific antigenic peptides to stimulate anti-tumor immune response (66). In conclusion, exosomes provide a powerful tool for different clinical purposes. Most of these applications will require further studies, but the results obtained so far pave the way for the promising application of exosomes in several clinical settings.

## Discussion

As the understanding of the physiological and pathophysiological functions of exosomes has increased, elucidating their role in a cancer setting has also gained momentum. The presence of the exosomes in different types of biological fluids make them accessible for screening with minimal invasiveness. Thus, exosomes isolated from patient-derived samples could act as potential diagnostic and prognostic markers of disease, which have rose excitement and interest among researchers. However, identifying such molecular markers with clinical utility remains a major challenge. Though multiple studies of exosomes and their cargo in various cancer types are being published (67–69), there are only few reports in the context of NB (42–44). Until now, there are only two published proteomic studies on NB-derived exosomes performed *in vitro* using cell lines (42, 43) and only one study carried out on the patient-derived samples (44) - thus limiting the translational applicability of exosomes to clinical settings. Therefore, more studies are required to reveal their role in aggressive metastatic phenotype.

The focus of this review is to explore the role of exosomal proteins in NB. It would be critical to delineate the individual and/or collective role of exosomal proteins and other functional biomolecules in metastasis. This is the area that requires immediate attention since monitoring and managing aggressive high-risk metastatic NB is challenging. Therefore, given the current knowledge and ease of collection and analysis of exosomes, their development as minimally invasive biomarker source for the disease diagnosis and prognosis holds great promise. This review gives an overview of NB exosomal protein studies and provides a starting point to facilitate future development of the substantive high-quality proteomics studies in this regard.

There are multiple methodologies used for the EV isolation, purification, and characterization. Due to this wide variety of available qualitative and quantitative methods, critical evaluation of these methods is necessary. For example, the size and concentration of EVs can be assessed by DLS, NTA, high-resolution flow cytometry (hFC) and tunable resistive pulse sensing (tRPS). Thus, data obtained by different set of instruments and settings can differ and significantly affect the corresponding results. Ultracentrifugation is the classical and most frequently used method for EVs isolation derived from biological fluids and cell culture supernatants. However, precipitation, ultrafiltration, immunoaffinity isolation, size exclusion chromatography and microfluidic techniques are also available. Each different method has its own advantages and disadvantages, which is important to consider when planning experiments. The ultracentrifugation method is cost effective, is suitable for the large volumes of samples and do not require additional chemicals. However, the method is time consuming, requires ultracentrifuge and has low yield or reproducibility. The precipitation methods are simple in operation and cost effective but could have potential contaminants. The ultrafiltration technique is simple, does not require special equipment or reagents but has filter membrane clogging issues. The immunoaffinity isolation technique has high specificity for exosome subtype isolation but its time consuming and expensive. The size-exclusion chromatography is known for its reproducibility, high sensitivity and scalability but requires specialize columns and has contamination issues. Finally, microfluidic technologies are rapid with good efficiency, but these are complex devices, requires additional equipment and expensive (70, 71).

Published reports show that researchers use different methodical approaches for exosome or proteomic studies (42–44). Different method of choice for exosome and proteomics preparations and analyses could affect the identifications of exosomal proteins. Therefore, care must be taken while choosing relevant methodology. Moreover, accurate estimation of sample contamination is a challenge and must be addressed with importance. Emphasis should be given to careful sample preparation both with respect to exosome and protein purification, identification, and validation. High-quality exosome preparations prior to downstream proteomic analyses are needed. The preliminary results highlighted in the present review should support future studies confirming the relevance of exosomal proteins in NB diagnosis/prognosis. To this end, the establishment of standard operating procedures (SOPs) will be essential to ensure data reproducibility and, eventually, enable the translation to a clinical setting.

The international society for extracellular vesicles (ISEV) have proposed minimal information for studies of EVs (“MISEV”) guidelines. It is important to consider the latest ISEV guidelines to report specific information (https://www.isev.org/misev).

Next, proteomic analysis of exosomes generates a large amount of data which is challenging to analyze. Appropriate bioinformatic and statistical analysis should be employed to analyze such extensive set of data. ExoCarta is an important resource of exosome proteomics studies (47) and should be explored to compare generated data.

Moreover, pre-clinical studies are also required to investigate the effect of potential exosomal proteins in animal model of NB. In this regard, multiple *in vivo* models including the subcutaneous and orthotopic xenografts derived from NB cell lines and patients-derived xenografts are available (72).

## Conclusion

Distant metastases remain the leading cause of NB mortality in children (7), which requires immediate attention. Better and improved knowledge in understanding the varied molecular mechanisms of metastasis is of great interest for the better cancer treatment. With the advent of exosomes-based therapies, the aim would be to generate novel, accurate, efficient, minimally invasive, and less-toxic treatment approaches. The rising interest in the exosome-based translational research will assist in the development of alternative treatment opportunities for children with high-risk metastatic disease. The use of exosome-based biomarkers for disease diagnosis and prognosis would be important as preventive treatment. This strategy would significantly decrease the dosage and duration of drug exposure and reduce the toxic side-effects caused by the conventional NB chemotherapy drug regimens. Thus, more research in this domain would improve the therapeutic possibilities and life expectancy of the patients with NB.

## Author contributions

SB: Conceptualization, Investigation, Writing – original draft, Writing – review & editing. MM: Writing – review & editing.
